# Gold nanoparticle transport across tumour-associated biological barriers: *in vitro* models, imaging, and quantification

**DOI:** 10.1039/d5nr02045j

**Published:** 2025-12-01

**Authors:** Christina Christodoulou, Alexander J. MacRobert, Marilena Loizidou, Bala Ramesh, Kate Ricketts, Christopher Thrasivoulou, Gavin Jell, Hirak K. Patra

**Affiliations:** a Department of Surgical Biotechnology, Division of Surgery and Interventional Science, University College London, Royal Free Hospital Pond St London NW3 2QG UK hirak.patra@ucl.ac.uk; b Division of Surgery and Interventional Science, University College London Charles Bell House 43-45 Foley Street London W1 W 7JN UK; c Division of Bioscience, Centre for Cell & Molecular Dynamics, University College London 21 University Street London WC1E 6DE UK

## Abstract

Gold nanoparticles have long been explored for their potential in medical diagnostics, drug delivery, and imaging, particularly in oncology. However, successful translation to clinical applications requires a deep understanding of their biomolecular interactions and transport mechanisms across cellular barriers and within cells. In this review, we examine the current understanding of the journey of gold nanoparticles from systemic administration to tumour infiltration. Specific focus is placed on the biological barriers crossed and the mechanisms involved in traversing those barriers, including active and selective transport pathways, like transcytosis, increasingly recognised as critical for nanoparticle translocation across endothelial and tumour barriers. We stratify the nanoparticle journey into smaller stages and critically discuss the most relevant *in vitro* models used to study each stage in isolation. Although traditional 2D cell cultures have provided some fundamental insights, more advanced tissue culture models outlined in this review offer enhanced physiological relevance. Monitoring nanoparticle behaviour within these models cannot be achieved without sophisticated imaging and quantification techniques. Herein, we have identified the most appropriate detection methods and their suitability for being used on each *in vitro* model for the detection of label-free gold nanoparticles. Using label-free nanoparticles preserves their native physicochemical properties and avoids potential artefacts introduced by fluorescent or radioactive tags, and conveniently, gold lends itself well to label-free detection due to its unique optical and electronic properties. By integrating insights from advanced *in vitro* modelling and cutting-edge detection strategies, this review highlights the current landscape and future directions for optimising the study of gold nanoparticle delivery across barriers in cancer nanomedicine.

## Abstract 2

This review uniquely combines gold nanoparticle transport mechanisms, advanced *in vitro* models, and label-free detection to guide translational nanomedicine research.

## Graphical abstract

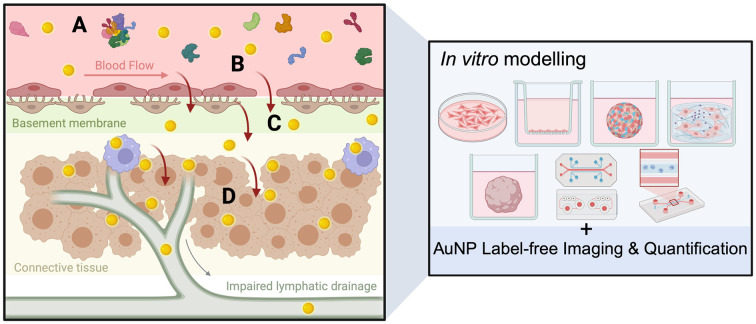

## 1. Introduction

Nearly three decades after nanomedicine research gained popularity, its impact is yet to be reflected in clinical practice. Nevertheless, nanomaterials continue to be widely researched for their therapeutic and diagnostic potential. Defined by the European Commission as materials with at least 50% of particles having one or more dimensions in the 1–100 nm range (recommendation 2011/696/EU), nanomaterials exhibit unique attributes at this scale that make them well-suited for drug delivery. Their ability to improve site-specific targeting aims to address the issues arising from non-specific, systemic drug distribution: adverse effects on healthy tissue, insufficient drug concentration at the disease site, and the incidence of drug resistance. Advances in nanotechnology have produced hundreds of different versions of nanoparticles (NPs), ranging from polymeric NPs to liposomes and metal NPs – each sharing common nanoscale traits but differing in their core composition and behaviour.

Gold is one of the earliest nanomaterials considered for medicine, with its use reported since ancient times as a red liquid or “liquid gold” used for longevity and disease treatment.^1^ Although gold nanoparticle (AuNP) use predates any mechanistic understanding, it demonstrates their low toxicity and chemical inertness. It is now established that gold nanoparticles (AuNPs) have nanoscale physicochemical properties that make them excellent candidates for acting as molecular carriers and radiosensitisers. These properties arise from the unique electronic characteristics introduced when gold is reduced to the nanoscale. At dimensions below 100 nm, especially below 10 nm, the restricted motion of electrons leads to altered optical, electronic, and catalytic properties. In ultrasmall gold nanoparticles, typically <2 nm, quantum confinement effects emerge, resulting in discrete energy levels and behaviours distinct from bulk gold, for example, inherent fluorescence.^2^

The atomic properties and electron profile of AuNPs introduce unique advantages compared to other NPs, evident in the versatility of their applications. Their ability to absorb radiation allows them to enhance radiotherapy, as it leads to the emission of secondary electrons that cause intense local DNA damage at the disease site.^3^ Also, by absorbing light, they can be used in photodynamic therapy, where they activate a photosensitiser that generates reactive oxygen species (ROS) which cause cell death.^4,5^ They can also be used in photothermal cancer therapy where they convert near infrared (NIR) light to heat, killing local cancer cells,^6,7^ typically involving AuNPs larger than 20 nm or anisotropic nanostructures (*e.g.*, rods, shells, stars) that exhibit plasmon resonance in the NIR range.^8^ A common application of nanosystems is as vectors for drug delivery, with hopes of targeting therapies to the disease site. The ease of functionalisation of AuNPs allows the conjugation of drugs and targeting moieties on the nanoparticle surface, providing the potential to guide NPs to specific cells in the body, for example for targeted chemotherapeutic delivery and targeted radiotherapy enhancement.^9^ Comprehensive discussions of AuNP conjugation and functionalisation strategies can be found in recent reviews.^10,11^ Additionally, as with other heavy metal NPs, their high electron density enables them to be used as a contrast agent in X-ray based imaging, magnetic resonance imaging (MRI), computed tomography (CT), positron emission tomography (PET) and other nuclear imaging techniques.^12,13^ A single agent could, therefore, be used for simultaneous treatment and diagnosis, referred to as ‘theranostics’.

Another reason why they have been studied so widely over the years is that colloidal AuNPs provide very stable systems and can be very easily synthesised. Several controlled chemical methods are available that can synthesise AuNPs of precise sizes spanning across the nanoscale, from 1 nm to over 100 nm.^14–17^ Since their properties are inextricably linked to their size, this precise control over their synthesis makes them highly functionally tunable.

Due to all the above reasons, functionalised AuNPs have been portrayed as promising for biomedical applications since the early 2000s, as summarised in a popular review from 2011.^18^ Unfortunately, despite the presence of 14 530 publications,††PubMed search: (“gold nanoparticle*”[Title/Abstract] OR “AuNP*”[Title/Abstract] OR “gold nanostructure*”[Title/Abstract] OR “gold nanomaterial*”[Title/Abstract]) AND (“medicine”[Title/Abstract] OR “targeted delivery”[Title/Abstract] OR “targeting”[Title/Abstract] OR “targeted therapy”[Title/Abstract] OR “therapy”[Title/Abstract] OR “tumour”[Title/Abstract] OR “tumour”[Title/Abstract] OR “cancer”[Title/Abstract] OR “cellular”[Title/Abstract] OR “biomedical”[Title/Abstract] OR “therapeutic”[Title/Abstract] OR “diagnostic”[Title/Abstract] OR “drug delivery”[Title/Abstract] OR “bioimaging”[Title/Abstract] OR “biosensor*”[Title/Abstract] OR “medical application*”[Title/Abstract]). there has been limited translation. Although some FDA-approved NP-based therapeutics have reached the clinic and had substantial impact, these are largely degradable nanosized carriers designed to protect biologically sensitive cargo, for example, lipid nanoparticles encapsulating mRNA in the Pfizer/BioNTech and Moderna COVID-19 vaccines, rather than actively targeted NPs directed to specific cells or tissues. The main causes of failure in actively targeted NP clinical trials are low efficacy, often reflected in only modest improvements in survival, and off-target toxicity.^19,20^ Also, to date, no AuNP formulations have been FDA-approved for therapeutic applications, even though AuNPs as colloidal-gold labels are widely used in FDA-cleared *in vitro* diagnostic lateral-flow assays,^21^ including OTC pregnancy tests (*e.g.*, K240242), respiratory virus antigen tests (*e.g.*, RSV, K132456), faecal occult blood tests (*e.g.*, K170548), and recent SARS-CoV-2 antigen assays (*e.g.*, K243518).

This might appear surprising considering the large number of *in vitro* and *in vivo* studies on NP-based therapies that show promise, including a high targeting efficacy^22,23^ and low toxicity.^24^ This lack of translation of results may in part be due to the inadequacy of *in vitro* models in mimicking the *in vivo* human environment. One example is the formation of a protein corona around the NPs, which may mask targeting moieties, and cause immunorecognition or other undesired interactions with off-target cells *in vivo*. These changes occur in ways that are difficult to predict and replicate across species, due to the variance in serum protein composition between species and individuals, complicating extrapolation from preclinical data.^25^ Furthermore, intravenously injected NPs must cross a number of biological barriers, including the vascular endothelium, other organ membranes, extracellular matrix (ECM), tumour microenvironment, cell membrane, and exosome membranes to reach their target. These features are not accurately represented in *in vitro* models, often resulting in suboptimal therapeutic concentrations at target sites. Importantly, species differences in these key barriers and animal physiology can account for the limited success in humans compared to mouse studies, as discussed in the next section.^26,27^ It is also reported that NP targeting may accelerate antigen depletion through receptor internalisation and degradation, which means that targeted NPs diminish their own targets.^28^ In order to enhance NP penetration across barriers, it is imperative to uncover the fundamental interactions between the NPs and biological systems. Identifying and studying the pathways naturally followed by NPs in the body will reveal the most effective strategies, for example, the type of functionalisation, that will harness said pathways to direct the NPs through the barriers to the desired location.

Integral to understanding AuNP biological interactions are the characterisation approaches used to image and quantify these interactions. Imaging objects in the nanoscale is tricky and often time-consuming, due to diffraction limits, low signal intensity, and resolution constraints in conventional microscopy techniques. As a result, many studies employ external fluorescent moieties to facilitate imaging. This in itself comes with its own limitations due to altering the NP properties (*e.g.* size and surface chemistry), but fortunately, AuNPs possess unique properties that, when harnessed correctly, can overcome the need for external labelling. Additionally, many available imaging and quantification techniques inherently destroy the sample, which prohibits its use in subsequent analyses and continuous monitoring. Quantitative determination of NP uptake, which is crucial for comparing different NP systems, is often acquired at the expense of spatial information. Therefore, it follows that both qualitative and quantitative methods need to be used in concert to obtain a comprehensive understanding of NP cellular interactions.^29^

**Fig. 1:**
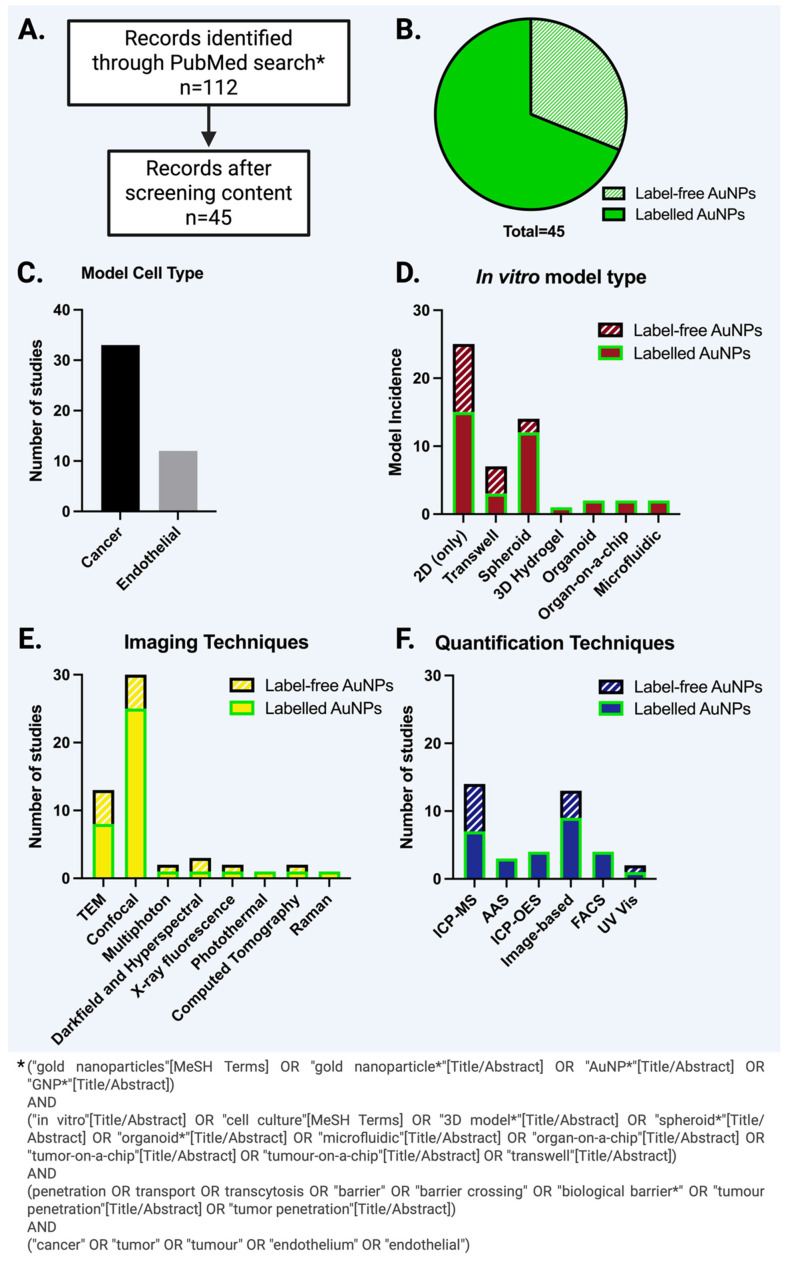
Summary of *in vitro* studies (October 2020–October 2025) investigating gold nanoparticles (AuNPs) in tumour-associated barrier crossing. (A) Overview of the literature selection process: 112 records were identified through PubMed, and 45 studies were retained after content screening. From these, we recorded: (B) the distribution of studies employing fluorescently labelled *versus* label-free AuNPs, (C) the cell types used in the barrier models (cancer cells for tumour penetration or endothelial cells for endothelial barrier studies), (D) the *in vitro* model types used in these studies, and (E and F) the imaging and quantification techniques employed, categorised by whether the AuNP systems were labelled or label-free.

To illustrate the current methodological landscape, we compiled and analysed recent studies identified through a structured PubMed search, summarising the *in vitro* models employed, the imaging and quantification techniques used to detect AuNPs, and whether external labelling strategies were applied (Fig. 1). This revealed recurring methodological limitations in current NP studies across barriers, with 20% of studies drawing conclusions without any quantitative measure, and 29% relying solely on image-based quantification, which can be unreliable due to limited sampling, subjective thresholding, optical artefacts, and the difficulty of converting signal intensity into accurate particle number or mass. Additionally, 69% of studies used AuNPs labelled with an external, usually fluorescent, moiety to aid detection, which has its own downsides, as discussed later. In the final sections of this review, we present tables outlining both commonly used and emerging techniques, together with brief descriptions of their suitability for different *in vitro* models of AuNP barrier crossing, ranked according to their sensitivity for label-free AuNP detection.

It is evident that, despite 25 years of research, our understanding of AuNP uptake and mechanisms of migration across biological barriers remains incomplete. To bridge the translational gap, future investigations require both robust and representative *in vitro* models, as well as reliable methods for temporally and spatially tracking the NPs. In this review, the journey of AuNPs through biological barriers is stratified into stages, and the suitability of models available at each stage is critically discussed. We follow the journey of AuNPs from the blood into a solid tumour, summarising the *in vitro* models currently in use for the investigation of NP migration, specifically across the endothelium and through a solid tumour. We also outline the current techniques available for label-free AuNP imaging and quantification in such models.

Interrogating the journey of AuNPs across cellular barriers in *in vitro* models will not only increase our understanding of AuNP behaviour but it will also enable the potential extrapolation of these findings to other types of nanoscale objects. Several nanosystems behave in similar manners by virtue of their size alone, which is why gold can be used as a valuable tool for nanomedical investigations considering its ease of synthesis, tunability, and detection.

## 2. Nanoparticle migration across biological barriers: *in vitro* models

The use of *in vivo* models is associated with a number of limitations, including cost, ethical concerns, long experimental times, and, importantly, species-specific biological interactions. For example, different species have different serum protein compositions, and some serum proteins, like albumin,^30^ have different levels of amino acid sequence conservation between species, which will influence protein corona formation.^31^ There are also significant differences in physiological architecture, like endothelial structure and tumour blood flow rate, which directly influence the extent of passive NP movement through tumour blood vessels, known as the enhanced permeability and retention (EPR) effect.

Several mouse studies demonstrate the leakiness of tumour vessels, observations including increased FITC-dextran leakage,^32^ interendothelial gaps of approximately 1.7 μm size,^33^ and abnormal, loosely associated basement membrane (BM).^34^ However, pioneering work by Warren Chan's group revealed that human interendothelial gaps are not as pronounced, accounting for the decreased EPR effect observed in humans.^35^ Additionally, human blood flow rate is about 810 times that of a mouse,^36^ something that is often overlooked when extrapolating observations in mouse models and scaling them up to humans.

When wanting to zoom in on a single interaction during NP transport, like migration across a biological barrier, *in vivo* models are far too complex to discern specific details in depth, as they bring numerous other variables into play.^37^ Simplified *in vitro* models mimicking a specific part of a biological process are preferred. Consequently, there is a growing shift towards sophisticated *ex vivo* and *in vitro* alternatives – more representative than 2D cultures, and without the limitations of animal models.^38^

Additionally, *in vitro* models can be useful in providing training data for machine learning models that could predict NP behaviour faster and more efficiently.^39^*In vitro* systems, with their controlled environments and reduced number of biological variables compared to *in vivo* studies, offer an ideal platform for distinguishing the influence of individual NP parameters, such as size, shape, surface charge, and functionalisation. Artificial intelligence (AI) approaches have already been employed in nanotoxicity predictions,^40^ transcytosis kinetic rate constant calculations,^41^ and protein corona composition predictions.^42^

Therefore, different *in vitro* models have been developed for the study of NP interactions, some of which will be discussed in this review for their use in the different stages of the AuNP journey (Table 1). AuNP translocation across biological barriers has been studied in several organ models, including the lungs, gut, skin, and placenta, to determine the fate of nanomedicines upon entering the body.^43^ Solid tumour models are also important for modelling nanoparticle tumour penetration. The number of NPs that enter the tumour after extravasation, as well as the depth of penetration, are important predictors of drug effectiveness, so investigating these parameters *in vitro* is critical for translation and rational nanomedicine design.^44^

**Table 1:** Common models relevant to studying the journey of nanoparticles *in vitro*: (a) 2D models, (b) transwell models, (c) 3D spheroid models, (d) 3D hydrogel models, (e) organoids, (f) organ-on-a-chip systems, and (g) microfluidic vascular networks

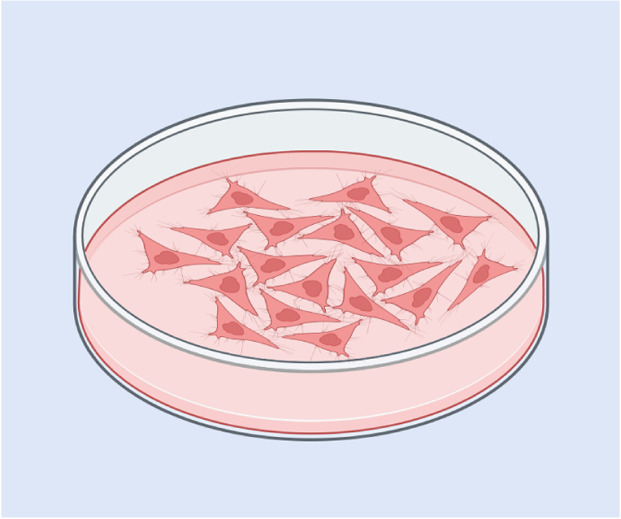	**(a) 2D cell cultures:** Traditionally, molecules were first tested on 2D cell cultures, the simplest type of *in vitro* model. In 2D cell culture models, cells are seeded as a monolayer, and although these models have been used to predict *in vivo* results, they lack the ability to recapitulate the tumour microenvironment
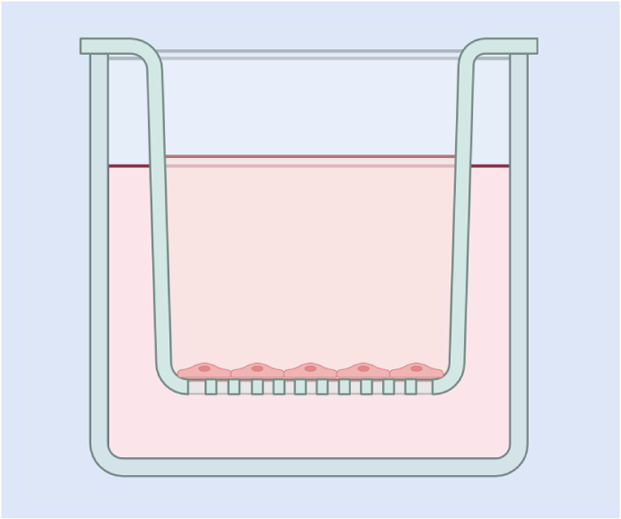	**(b) Transwell systems:** Widely used *in vitro* models consisting of two compartments separated by a permeable membrane. Cells are cultured on the membrane, allowing the study of nanoparticle transport, barrier integrity, and cellular interactions. Useful for modeling biological barriers, such as the endothelium, blood–brain barrier, and intestinal epithelium
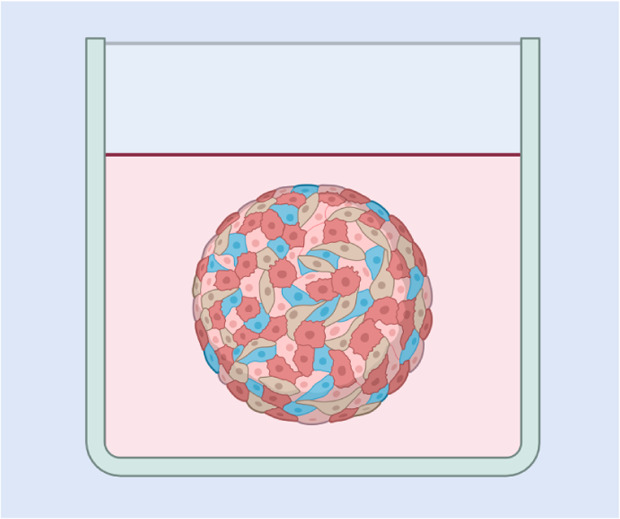	**(c) Spheroid models:** 3D aggregates of cells that mimic the architecture and microenvironment of solid tumors. They create gradients of oxygen, nutrients, and pH similar to those found *in vivo*, making them ideal for studying nanoparticle penetration, accumulation, and therapeutic efficacy within dense tumor tissues
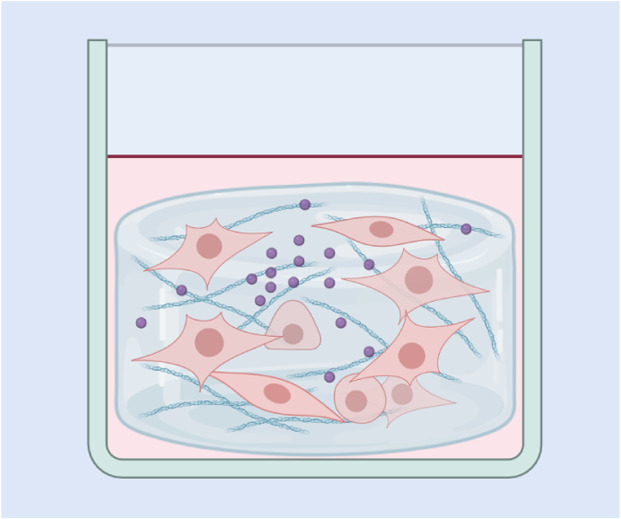	**(d) 3D hydrogel models:** These use biomimetic matrices, such as collagen or synthetic hydrogels, to replicate the extracellular matrix (ECM) of tissues. They support cell growth and allow investigation of nanoparticle diffusion, cellular uptake, and interaction with ECM components, closely mimicking the physical and chemical properties of *in vivo* environments
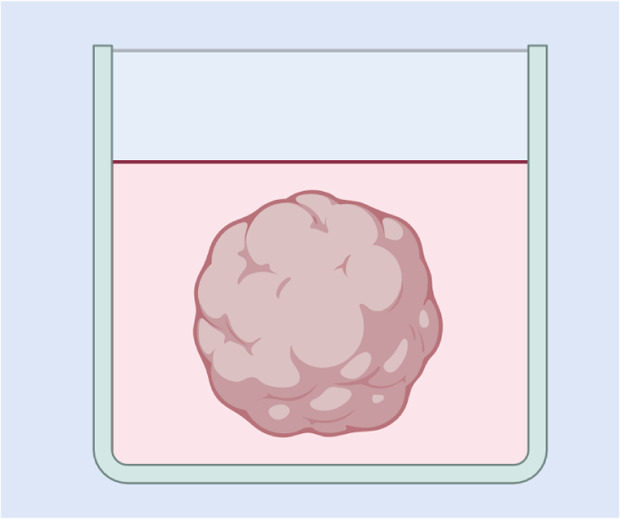	**(e) Organoids:** 3D structures derived from stem cells that self-organise into miniaturised versions of organs, replicating key aspects of their architecture and functionality. They provide a complex and biologically relevant environment for studying nanoparticle interactions, drug responses, and tissue-specific processes, such as tumour growth or barrier function
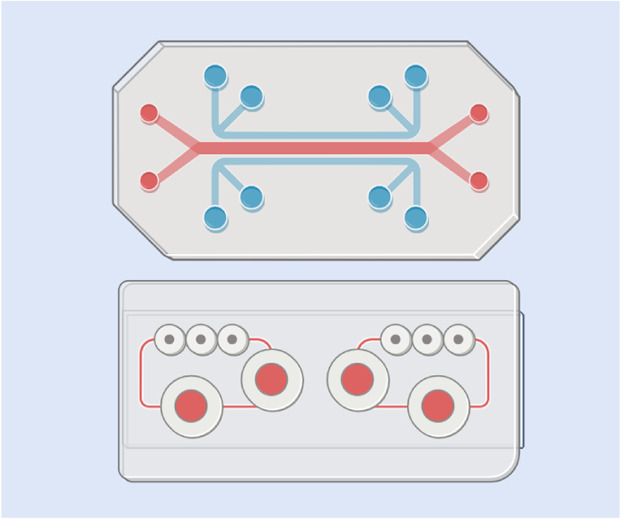	**(f) Organ-on-a-chip systems:** Microfluidic devices designed to replicate the structure and function of specific organs or tissues. These dynamic models incorporate multiple cell types under physiologically relevant flow conditions, enabling precise study of nanoparticle behavior, transport mechanisms, and drug delivery in organ-specific microenvironments
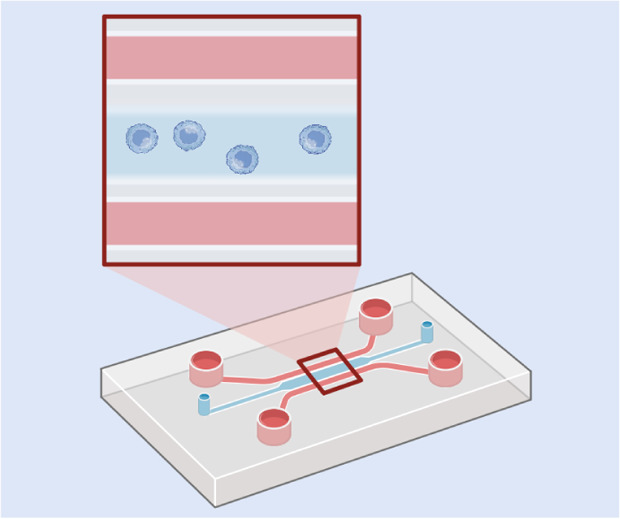	**(g) Microfluidic vascular networks:** Microfiuidic *in vitro* systems that simulate blood vessels and vascularised tissues. These models incorporate endothelial cells and mimic flow dynamics, providing a platform to study nanoparticle transport, adhesion, and interactions with the vascular endothelium under shear stress and physiological conditions

## 3. Modelling the nanoparticle journey

### 3.1. Entering the blood: protein corona formation

**Fig. 2:**
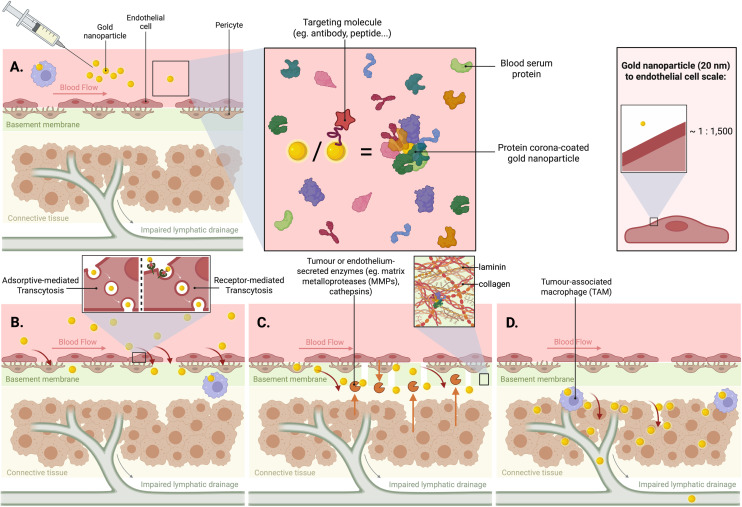
The journey of AuNPs from the blood to the cancer mass. (A) Intravenous administration of AuNPs and the formation of a protein corona by the interactions of the NPs with circulating blood serum proteins, increasing the effective size of the nanoparticle. (B) Transendothelial movement of NPs, either through the enlarged intercellular gaps of the leaky cancer vasculature, or by transcytosis through the endothelial cells. (C) Crossing of the BM surrounding the blood vessel to reach the (D) tumour microenvironment. Once in the connective tissue surrounding the tumour, they can bind to the cancer cell surface, be internalised by the cells, and then penetrate deeper into the solid mass, through transcytosis.

Upon administration in the blood of a living organism, or in the culture media of an *in vitro* model, the NPs are immersed in a fluid of complex composition. The NP's surface chemistry determines its interactions with water and the surrounding biomolecules, leading to the formation of the protein corona which forms almost instantaneously around its surface^25^ (Fig. 2A). Although traditionally overlooked, the influence of the protein corona is being increasingly recognised, as it can dramatically alter NP physicochemical properties, including effective size and charge, cellular uptake,^45^ and *in vivo* circulatory duration and excretion rate.^46^ Some proteins bind more tightly than others, sometimes referred to as the “hard corona”, while others bind more loosely and are in a more dynamic equilibrium with the surrounding biological medium, referred to as the “soft corona”.^47^

Being the first point of contact, the protein corona can significantly influence NP recognition and uptake by immune cells. In many cases, if it's rich in opsonins like complement proteins or immunoglobulins, the protein corona can enhance phagocytosis through the activation of receptor-mediated uptake pathways in macrophages.^48^ On the other hand, a corona that is rich in dysopsonins like albumin can mask the NP surface and protect it from the foreign body host response. A study has found that NPs pre-incubated in human serum were shielded from macrophage uptake, enhancing immune evasion.^49^ This suggested that of those proteins that could bind macrophage receptors, very few had their motifs correctly exposed for binding.

Importantly, for nanomedical formulations, the protein corona can mask targeting moieties conjugated on the NP surface for specific attachment to target cells, including targeting peptides or antibodies. Binding reductions of 94% and 99% were observed when bicyclononyne-targeted NPs were exposed to 10% and 100% serum mediums, respectively, showing a significant decrease in targeting efficacy.^50^

These insights have inspired research on the strategic design of NPs in order to predict and control the protein corona composition, or limit its formation.^51^ Attempts have been made to limit protein corona formation with the aim to prolong nanomedicine half-life, as reviewed by Rampado *et al.*,^52^ with the most popular example being the use of polyethylene glycol (PEG). PEG imparts a highly hydrophilic surface chemistry to NPs, effectively reducing non-specific protein adsorption by promoting strong interactions with surrounding water molecules. It has been found that coating NP surfaces with molecules like PEG and PCS glutathione-S transferase can limit corona formation and subsequently reduce their uptake by macrophages.^53^ However, due to the diverse polar and structural properties of plasma proteins, complete inhibition of protein binding is rarely achieved. Despite the established role of PEG as a stealthing agent, recent work has detected the presence of increased anti-PEG antibodies in the protein corona of PEGylated nanocarriers, which increases uptake by macrophages, accelerating blood clearance.^54^ It was also found that pre-coating NPs with a recombinant fusion protein before immersing them in 55% foetal bovine serum (FBS), led to an increased uptake by the target cancer cells and a decrease in macrophage uptake *in vitro*.^53^ Such studies highlight the potential benefits of intentionally predefining the protein corona by pre-coating NPs with specific proteins or polymers. This offers improved control over subsequent protein adsorption, a process influenced by the Vroman effect,^55^ which describes how initially adsorbed, more mobile proteins are progressively replaced by less mobile proteins with higher surface affinity. However, such pre-coating does not fully prevent this dynamic exchange of proteins on the NP surface.

#### 3.1.1. Modelling the protein corona

The protein corona can be recapitulated *in vitro* through the addition of blood serum in cell culture media, as is normally done in typical cell culture. Although this is a simple approach, achieving a representative protein composition is far from trivial. Serum differences can be a major cause of discrepancy between protein corona compositions and thus NP behaviour in *in vitro* models compared to *in vivo* studies and compared to the clinic.

Serum differences *in vitro* can arise either due to the use of different serum species or even different batches of the same serum which can differ in protein composition. FBS is the most commonly used serum to supplement cell culture media, however, its composition is not very representative of human blood serum.^31^ It has been shown that the amount of NP-bound proteins is twice as high after incubation in HS compared to FBS *in vitro*,^56^ but the influence of the corona on NP uptake remains poorly understood. This problem doesn't stop at *in vitro* modelling, but the same limitation applies to *in vivo* studies. Protein coronas formed in human plasma and those formed in mouse plasma showed significant differences in their proteomic profiles – the corona from human plasma was enriched with immunoglobulins and complement proteins, while that from mouse plasma contained higher levels of fibrinogen and serotransferrin.^57^

Since animal sera have demonstrated clear differences in protein corona formation, human serum (HS) is speculated to be more suitable for supplementing tissue culture media *in vitro*. However, beyond inter-batch and inter-donor variability, a critical yet often overlooked limitation lies in the pathological alterations of blood composition in patients – the very individuals nanomedicines are intended to treat. Conditions such as cancer, chronic inflammation, blood conditions, or even pregnancy, as well as lifestyle choices like smoking, can inherently alter plasma protein profiles,^58^ affecting the protein corona composition and influencing key aspects of the NP journey discussed above. This is not reflected in HS-supplemented *in vitro* models since the serum comes from healthy donors. Theoretically, the closest to replicating a diseased state *in vitro* would be through the purification of patient-specific serum for personalised studies; however, this is a resource-intensive and time-consuming task. Additionally, more subtle inter-patient variability, such as the use of medications or individual physiological responses, could further complicate the reproducibility and generalisability of results.

While taking into account the importance of the protein corona in *in vitro* models is important, their interpretation ultimately depends on robust experimental characterisation. The protein corona is studied using a range of complementary imaging and analytical techniques. Mass spectrometry-based proteomics (*e.g.*, LC–MS/MS) is the primary approach for identifying and quantifying adsorbed proteins, often following SDS–PAGE or 2D electrophoresis.^59^ However, recent multi-laboratory studies by the Mahmoudi group revealed substantial variability in protein-corona proteomic profiles obtained from identical samples across independent laboratories, underscoring the need for standardised experimental and data-processing pipelines to ensure reproducibility across facilities.^60–62^ Dynamic light scattering (DLS) and zeta potential measurements can rapidly indicate corona formation *via* changes in particle size and surface charge,^63^ while isothermal titration calorimetry (ITC) and surface plasmon resonance (SPR) provide binding affinities and kinetics. Additionally, electron microscopy (TEM, cryo-EM) can reveal structural features,^64^ while super-resolution fluorescence microscopy has enabled direct visualisation of the protein corona on single NPs.^65^ More recently, advanced analytical methods, some of which label-free (*e.g.*, Raman, infrared spectroscopy, nanoparticle tracking analysis), have enabled *in situ* probing of corona dynamics at the single-particle level. Notably, new single-particle optical methods such as RONAS (real-time optical nanoparticle analysis by scattering microscopy) have enabled label-free tracking of corona formation directly in complex biological fluids.^66^

### 3.2. Crossing the endothelium

Regardless of immune cell uptake or immune evasion following intravenous administration, the NPs will inevitably have to cross the vascular endothelium to achieve extravasation into the target tissue or clearance (Fig. 2B). Before reaching the cells, the NPs first interact with the glycocalyx, a dense, gel-like layer of sugars and proteins of approximately 400 nm thickness that coats the luminal surface of endothelial cells.^67^ This is the initial area of adhesion before extravasation, and it significantly affects the fate of the NPs, as its disturbance was shown to increase NP movement *in vitro*.^68^ Lastly, underneath this gel-like layer is the main barrier: the endothelial cell layer. It is crucial to understand the mechanistic details of NP transendothelial movement, which may occur either intercellularly, through the cellular junctions, or transcellularly.

It was thought that NPs in the bloodstream mainly entered the interstitial space through gaps between the cells in the vascular endothelium by a combination of diffusion and convection,^69^ a process governed by pressure differences described by Starling's law.^70^ In the 1980s, NP extravasation was interestingly observed to be more pronounced in the tumour vasculature compared to other blood vessels, which suggested that NPs might have an intrinsic selectivity towards cancer tissues, reported by numerous authors including Matsumura, Dvorak, and Muggia *et al.*^32,71,72^ This phenomenon, named the Enhanced Permeability and Retention (EPR) effect, naturally made NPs gain popularity in the field of targeted cancer treatment and diagnostics. It was rationalised by cancer tissues having disorganised, leaky blood vessels with deficient basement membranes (BMs),^33^ as well as lacking a functional lymphatic system for efficient clearance of the NPs.^73^ Also thought to contribute to the elevated vascular permeability were factors actively produced by tumour cells, including bradykinin,^74,75^ nitric oxide (NO) and peroxynitrite (ONOO–)^76^ and vascular endothelial growth factor (VEGF),^77^ as well as other inflammatory mediators. Taken together, these would make NPs more likely to extravasate through tumour vessels and accumulate in the tumour microenvironment compared to healthy tissue.

Despite signs of favourable accumulation in tumours *in vivo*, clinical results suggest otherwise. The lack of success in the clinic, as discussed earlier, could be due to a discrepancy between tumour physiology in animal models and human tumours, and the heterogeneity between different tumour types, making the EPR effect more pronounced in mice, for example, than in humans.^78^ A study measuring the number of gaps seen in human tumour blood vessels found the overall gap coverage to be 0.048% of the blood vessel surface area, with most of the gaps being transcellular channels rather than intercellular gaps.^35^ In fact, even *in vivo*, results have shown that very few NPs actually reach their target. A *meta*-analysis from 2016 concluded that only 0.7% of the NPs administered reached solid tumours in *in vivo* studies,^27^ and one from 2020 found a median 0.76% delivery efficiency in 200 mouse studies subjected to physiologically based pharmacokinetic (PBPK) modelling and simulation analyses.^79^ Such studies, coupled with the insufficiency of recorded clinical benefits conferred by NP-based therapies, have led to the significance of the EPR effect being debated,^26^ with some researchers even suggesting that the effect is simply an experimental artefact. More recent critical analyses emphasise that the EPR effect is highly heterogeneous across tumour types, often absent in human cancers, and cannot be relied upon as a universal mechanism for drug delivery. This has prompted calls for a paradigm shift in NP design, focusing less on passive EPR-driven accumulation and more on active, targeted, or transcytotic transport pathways.

Evidence is now suggesting that about 97% of NP movement is transcellular as opposed to intercellular.^35^ Transcellular movement can involve passive diffusion through the endothelial cells, movement through intracellular fenestrations, or transcytosis, which includes sequential endocytosis, intracellular vesicular trafficking, and exocytosis^19^ (Fig. 2B). Research has also suggested the presence of a subpopulation of endothelial cells that showed significantly enhanced AuNP uptake, named NP transport endothelial cells (N-TECs). Differential gene expression analysis of N-TECs compared to the rest of the endothelial cells has revealed the upregulation of genes associated with the clathrin-mediated endocytosis pathway as well as genes mediating microvascular permeability and transcytosis.^80^

If NP extravasation through the EPR effect proves insufficient for effective tumour infiltration, innovative strategies need to focus on leveraging active transcellular pathways for NP transport. Transcytosis combines elements of endocytosis with processes that deliver proteins to specific cell surfaces, offering a selective and fast mechanism for transporting vesicles from one pole of the cell to the other.^81^ The term was first introduced by N. Simionescu to describe the transport of macromolecules within plasmalemmal vesicles from the bloodstream to the tissue interstitium, across the capillary endothelium.^82,83^ Transcytosis is naturally involved in the transport of various biological molecules, including antibodies across the intestinal epithelium into the gut lumen, proteins and antibodies across cellular barriers,^84^ molecules across the BBB,^85^ and even viruses like HIV across epithelial cells.^86^ By engineering NPs to exploit transcytosis, for example through the conjugation of transcytosis-enhancing peptides or small molecules, researchers can enhance and potentially target NP trafficking for more efficient delivery. An example is the use of RGD peptides which bind integrins αvβ3 and αvβ5 overexpressed on the tumour endothelium,^87^ famously iRGD, which has shown a lot of promise in transcytosis enhancement.^88^

One special type of endothelium on which a lot of research is focused is the blood–brain barrier (BBB), the endothelial barrier that separates the blood from the brain parenchyma. Notorious for its highly selective permeability, the BBB presents a major challenge for nanomedicine delivery, as its tight junctions, low transcytosis rates, and active efflux transporters prevent most therapeutics from reaching the brain. This poses a significant hurdle in the treatment of neurological disorders and brain cancers, where effective drug delivery is critical. Due to its tightly packed cells preventing paracellular transport, efforts focus on employing transcytosis pathways for BBB crossing, for example by adding transferrin to AuNPs to target transferring receptors.^89^ There are also several synthetic peptides that target BBB receptors including the nicotinic acetylcholine receptor, transferrin receptor, low density lipoprotein receptor-related protein 1 (LRP-1) and low density lipoprotein receptor (LDLR).^90^ Several NP systems have been functionalised in different ways that facilitate crossing the BBB, as recently reviewed by Zha *et al.*^91^

#### 3.2.1. Modelling the endothelial layer

Replicating the complexity of the endothelial layer remains challenging due to its dynamic physiological functions, tissue-specific heterogeneity, and sensitivity to mechanical and biochemical cues. In the past, vascular endothelia were modelled primarily using 2D cultures; however, newer and more complex models have been developed to better capture human blood vessel structure. These include transwell models, 3D hydrogel models, and more complex microfluidic vascular models (Table 1).

One common *in vitro* model designed to mimic biological barriers is the transwell model, which involves the growth of a cell monolayer on a permeable insert that separates the system into two compartments.^92^ Endothelial cells are seeded onto the permeable support within the insert, forming the upper compartment of the model that represents blood contents (Table 1b). The permeable support is often coated with protein layers before cell seeding, such as laminin, collagen, or gelatin, to enhance cell adhesion and mimic the extracellular environment. Drugs or NPs are typically introduced into the upper compartment, simulating intravenous administration, to study their transport across the endothelial barrier. Other cell types can be seeded either at the bottom of the well underneath the cell insert, or on the underside of the permeable support, forming non-contact and contact co-culture models, respectively. In non-contact co-culture models, soluble factors secreted by the cells at the bottom of the well can diffuse through the medium, inducing and activating the endothelial cells above. These models also allow for easy separation of the two cultures for further downstream analysis. In contrast, contact co-culture models involve direct interaction between the two cell types, which can represent some biological situations. However, physical contact makes their separation for further analysis more difficult.^93^

Endothelial layers have also been modelled using 3D collagen hydrogel scaffolds. In this approach, endothelial cells are embedded within a collagen hydrogel, where they gradually migrate to the ventral side of the scaffold over a 14-day culture period. This process leads to the formation of an endothelium-like cellular layer on the surface of the gel, mimicking the structure of natural endothelial barriers.^94^

Microfluidic vascular networks are also used for modelling endothelial layers, closely replicating the structural and functional characteristics of blood vessels *in vitro*.^95^ These networks consist of microchannels lined with endothelial cells, mimicking the natural architecture, shear stress, and dynamic flow conditions present in the vasculature. Unlike static models, microfluidic systems allow for precise control over fluid flow, nutrient exchange, and biochemical gradients, enabling the study of endothelial behaviour in a physiologically relevant environment. Such systems have been engineered using a range of techniques, such as needle-based channel formation, sacrificial molding, bioprinting, and micropatterning, as reviewed by Hasan *et al.*^96^ Additionally, microfluidic vascular models facilitate high-resolution imaging and real-time monitoring of cellular interactions, favouring their application in NP transport.^97^

While similar modelling methods can be used, the BBB's unique characteristics call for specialised versions of endothelial models, which may include co-culturing additional cells present at the BBB, such as astrocytes. Transwell models are the most prevalent and widely studied for modelling the BBB, often in a contact co-culture with astrocytes.^93,98^ An *in vitro* BBB spheroid model was also developed using six brain cell types: astrocytes, pericytes, endothelial cells, microglia, oligodendrocytes, and neurons.^99^ Tri-culture BBB-on-a-chip models using endothelial cells, pericytes, and astrocytes have also been developed, enabling NP quantification by fluorescence intensity measurements and cell-type–specific uptake *via* FACS, as well as permeability coefficient (*P*_e_*P*_a_*p*_p_) extraction and TEER measurements for barrier integrity confirmation.^100^

Lastly, other than the way the cells are arranged to build these different types of models, another important consideration is the type of cells included. The endothelia of different organs, as well as endothelial cells derived from different vascular structures (for example veins, capillaries, or arterioles) have demonstrated differential AuNP uptake, in both *in vitro* models and their *in vivo* biodistribution.^101^ This is likely attributed to the anatomical and physiological diversity between the endothelia, and is another factor to consider when building a representative model. Human umbilical vein endothelial cells (HUVECs) are widely used primary endothelial cells for *in vitro* vascular studies with NPs, and are generally considered reliable for such models, as concluded in a review by Cao *et al.*^102^ A deeper understanding of the unique trafficking pathways across different types of endothelia holds significant potential for optimising the targeted delivery of nanomedicines to specific disease sites.

### 3.3. Entering the tumour microenvironment: traversing the endothelial basement membrane

After traversing across the cellular layer, the NPs encounter the endothelial basement membrane (BM), another critical barrier to NP delivery. It is a form of ECM composed mainly of laminin and collagen IV, as well as other molecules like entactin and perlecan.^103^ In healthy tissue, the BM closely surrounds the endothelial cells and pericytes, providing mechanical support and serving as a key filter to substances entering the interstitium. Additionally, the BM regulates cellular behaviours like differentiation, proliferation, adhesion, and migration.

In tumours, the BM separates the connective tissue and tumour cells from the bloodstream, making it a key defence mechanism against metastasis. Although the tumour BM is thought to be loosely associated with the surrounding cells and contain gaps and structural abnormalities, it was still found to be present in 92% of the tumour vasculature,^104^ suggesting that it's worth considering when investigating and modelling the endothelial barrier for cancer treatments. Tumour-secreted enzymes like matrix metalloproteinases (MMPs) and cathepsins can degrade the BM, with the goal of facilitating tumour cell invasion and metastasis,^105^ leading to gaps or weaker points in the membrane. This also facilitates the diffusion of NPs through it (Fig. 2C), however, NPs can still get trapped in the BM on their way to the tumour.

Some suggest that breaching the BM is essential for efficient NP-drug delivery to tumours.^106^ One proposed strategy to enhance NP delivery involves purposefully disrupting the BM using collagen hydrolases. While this technique could facilitate NP movement towards the cancer tissue, it also increases the risk of metastasis, as the compromised barrier may allow invasive cancer cells easier access to tumour blood vessels.^19,107^ On the contrary, malignancies associated with an already compromised BM might naturally enable easier passage of nanomedicines, making those patients more likely to benefit from such therapies.

#### 3.3.1. Modelling the basement membrane

The endothelial BM can simply be modelled as an additional component of the endothelial models, by incorporating some of its key components in the models. In simpler *in vitro* models, like 2D or transwell models, the cell culture surface can be coated with BM proteins prior to cell seeding. Commonly used components include collagen and laminin, which are the primary structural elements of the BM, as well as mixtures of these proteins to better mimic its complexity. Additionally, Matrigel – a murine-derived BM extract rich in laminin, collagen IV, entactin, and growth factors – is frequently used as a substrate to recreate a more biologically relevant microenvironment.^108^ Despite its representative composition, its undefined and non-human origin limits its physiological relevance, particularly for clinical translation, and the batch-to-batch variability in composition, stiffness, and growth factor content limits reproducibility.

There are also several methods for incorporating the BM in organ-on-a-chip models and microfluidic systems which have been thoroughly reviewed by Salimbeigi *et al.*^109^ Some include microfabrication techniques, bioprinting, and electrospinning, using either natural or biocompatible polymers like PDMS, PC, or PET.

Unfortunately, the limited data available on the biophysical and mechanical properties of the BMs of different organs, mainly due to the lack of isolation and characterisation techniques, makes it harder to recreate them *in vitro*. Addition of the BM as an extra feature of *in vitro* models will not only provide a more reliable recapitulation of the barrier, but will also add a physiologically relevant substrate that influences cell morphology and behaviour, such as migration, proliferation, and differentiation.

### 3.4. Penetrating through the tumour

Tumour-specific NP accumulation increases the local concentration of therapeutic agents at the disease site by allowing targeted release of cytotoxic cargo or radiation enhancement to the tumour. This can limit the incidence of adverse side effects on healthy tissue, which often restricts doses, offering an improved safety profile compared to traditional chemotherapy. Efficient targeting is beneficial not only for therapeutic purposes but also for diagnostic applications. Functionalised NPs can serve as imaging probes or biosensors, aiding in the early detection, monitoring, and characterisation of cancer.^110^ This dual therapeutic and diagnostic potential highlights the versatility of NP-based systems in oncology.

Delivery of NPs to tumours can be achieved through either passive or active mechanisms. Passive accumulation relies on non-specific extravasation from the systemic circulation, as discussed earlier, and then passive diffusion through the tumour microenvironment. Tumour infiltration by diffusion or convection is slow and is impeded by various physical parameters, for example, the high viscosity of protein-rich components like the ECM, the densely packed cellular environment, and the elevated interstitial fluid pressure (IFP) which reduces convective transport.^111^ Research has focused on the development of active targeting strategies, which can occur either at the level of the tumour endothelium or at cancer cells directly. The most common method involves conjugating receptor-specific antibodies or ligands to the NP surface, enabling them to selectively recognise and bind to cancer cells while sparing normal cells.^112^ AuNPs, with their highly modifiable surfaces, offer a versatile platform for active targeting. Their surfaces can be functionalised with a range of biomolecules, including antibodies, small molecules, and peptides, to enhance solubility, improve cell-specific uptake, and enable targeted delivery to cancer tissues.^113–115^

Antibodies are one of the obvious choices for surface targeting due to their remarkable specificity and diversity, making them valuable tools for directing NPs in drug delivery, imaging, and biosensing applications.^116,117^ This combination of functional versatility and precise targeting has positioned AuNPs as a promising technology for cancer diagnostics and treatment. While antibody-based targeting appears promising in theory, it has proven to be more complicated than anticipated *in vivo*. A study reported that only 0.0014% of Trastuzumab-coated AuNPs successfully interacted with cancer cells in tumours,^118^ despite Trastuzumab's well-established affinity for the HER2 cancer marker and its clinical efficacy as an FDA-approved therapy.^119,120^ This highlights the existence of poorly understood biological interactions that impede successful targeting. Possible justifications include NP sequestration by tumour-associated macrophages (TAMs) (Fig. 2D) and entrapment in the ECM.^118^ On a whole-organism scale, NPs face additional challenges, such as competition with the mononuclear phagocytic system (MPS) and renal clearance pathway, which prevent NPs from reaching the tumours.^121,122^

Furthermore, limitations in conjugation strategies, including instability or improper orientation of antibodies after conjugation, can adversely affect targeting efficiency.^123^ Proposed solutions for this include the use of adapters which properly position antibodies on the NP surface without obstructing antigen binding sites.^124^ In addition to facilitating active targeting, evidence has suggested that antibody conjugation may also increase the probability of NP uptake *via* endocytosis, improving tumour penetration.^125^ This indicates that successful targeting strategies could simultaneously overcome tumour penetration barriers, enhancing the overall efficacy of NP-based therapies.

Beyond effectively arriving at the tumour site after extravasation, which is the aim of the above surface targeting strategies, to exert their full effect, NPs must penetrate deeply into the solid tumour mass (Fig. 2D). The extent of drug penetration within tumours is a good predictor of efficacy, and adequate tumour cell eradication is needed to prevent the survival of regenerative cancer cells that could lead to recurrence. This implies the importance of ensuring that therapeutic NPs not only accumulate near the tumour site but also infiltrate deeply and uniformly within the tumour tissue.^126^ This can occur transcellularly by actively crossing tumour cellular barriers rather than passing between cells. In this process, known as transcytosis, NPs are taken up by cells and then expelled, in an endocytosis–exocytosis-like manner, resembling mechanisms observed in endothelial cells.

Transcytosis, typically used for the transport of macromolecules across the endothelium, could also facilitate the movement of NPs between adjacent cancer cells within a solid tumour. While it remains unclear whether NPs naturally exploit this pathway for tumour penetration, its potential for being manipulated for the enhancement of NP penetration is significant. As mentioned earlier, transcytosis has been successfully manipulated for the transport of NPs across biological barriers, such as the BBB^89,98^ and the gut epithelium.^127^ Several transcytosis-enhancing and cell-penetrating peptides, for example iRGD, which was mentioned earlier for tumour endothelium transcytosis, are also used for tumour penetration.^88^ This mechanism could be harnessed to achieve wider and deeper NP distribution within tumour tissues through transcellular migration.^128^

#### 3.4.1. Modelling solid tumours

To investigate the depth of NP penetration, the tumour environments can be simulated *in vitro*. These *in vitro* models are incubated with NP-containing media, mimicking the exposure of a tumour to therapeutic agents in the interstitial space. While simple 2D cancer cell cultures can be used for such investigations, 3D models provide a more accurate representation of the tumour microenvironment. These 3D models, often in the form of spheroids or organoids, offer varying levels of complexity and better replicate the structural and physiological characteristics of solid tumours.

Simple 3D *in vitro* models involve cancer cells cultured in a way that promotes the formation of spherical cell masses, known as spheroids. Spheroids can be formed by several methods, with or without scaffolds. One simple scaffold-free technique is the use of ultra-low attachment plates which prevent cell adhesion and encourage spheroid formation.^129^ In the hanging drop technique, which is also scaffold-free, cell suspensions rely on microgravity to concentrate the cells and form a mass at the bottom of a hanging drop.^130^ Magnetic levitation can also be used, where cells pre-treated with paramagnetic iron oxide NPs are seeded on low-adhesion plates and are exposed to a magnet, causing them to levitate and form spheroids.^131^ Most scaffold-based techniques usually involve the use of hydrogel scaffolds, which can be derived from natural ECM or synthesised. The high water retention capacity of hydrogels due to microscopic pores in the material enables the diffusion of soluble substances including oxygen, nutrients, and growth factors, necessary for cell viability.^132^ Matrigel, previously mentioned for BM modelling, is one of the most popular natural ECM-based hydrogels containing laminin, collagen, and other ECM components. Cells can be seeded on top of solidified Matrigel or suspended within the liquid gel for embedding, but as mentioned earlier, being murine-derived, it can introduce species mismatch limitations and batch-to-batch variability. For large-scale spheroid production, rotary cell cultures or bioreactors are often employed. These systems typically use stationary scaffolds within the culture flask to support spheroid formation.^133^

Organoids are *in vitro* 3D models, typically derived from stem cells, that closely mimic organ architecture, cellular heterogeneity, and microenvironmental interactions.^134^ Tumour organoids, that are usually made from patient cells, preserve key cancer traits, support long-term growth, and better replicate tumour complexity. These are often referred to as “tumouroids” and aim to more closely replicate the composition and microenvironment of solid tumours. Complexity can be increased by co-culturing additional cell types, including fibroblasts, endothelial cells, and immune cells, alongside matrix components.^135,136^ Additionally, careful control of key tumour properties can be achieved, such as monitoring oxygen levels, incorporation of a representative collagen content, or control of the overall density, as these factors directly influence NP movement through the tumour mass. To even better mimic tumour physical properties, tumouroid models that are embedded in hydrogel scaffolds can also be mechanically compressed.^136^ By bridging the gap between simple 2D cultures and complex animal models, 3D tumouroid models address many limitations associated with each, providing a more physiologically relevant platform for measuring NP uptake and penetration.^114^

A simpler model to investigate NP penetration across cancer cells is the transwell model. Although more commonly employed to study monolayers such as endothelia, transwell models can also be adapted for cancer research. In this setup, cancer cells are seeded on the permeable support of a cell culture insert, dividing the system into two compartments. The cells can be cultured to form multiple layers on the upper side of the membrane, mimicking the cellular layers of a solid tumour, and they can be incubated with NP solution.^128^ Measuring the NP concentration in the basolateral compartment could indicate how effectively they were able to pass through the cancer cell layer, mimicking penetration.

Vascularisation in the form of microfluidic models provides an additional level of physiological relevance by incorporating perfusable blood vessel networks, enabling the study of NP delivery within a controlled tumour-on-a-chip system.^137^ This allows precise control over fluid dynamics and NP transport, closely mimicking the conditions NPs encounter when penetrating a solid tumour mass.^138^ Tumour-on-a-chip models have allowed researchers to study NP diffusion, extravasation, and retention within the tumour microenvironment, providing valuable insights into drug delivery efficiency and therapeutic optimisation.^139,140^ For example, such models have been used to resolve ligand-dependent transcytosis and extravasation under physiological shear, and have shown how they can predict *in vivo*-like NP behaviour and dissect active transport contributions beyond the EPR.^139^ Recent reviews have comprehensively reported the different types of tumour-on-a-chip models of varying complexity, from single-compartment to multi-organ multi-compartment platforms, developed for studying NP behaviour.^141^

## 4. Mechanisms of gold nanoparticle cellular uptake

Given that NPs can interact and be taken up by various cells, understanding the mechanisms employed for uptake is crucial. NP uptake is desired when it occurs in target cells, but counterproductive off-target. To exert any control over which cells NPs are taken up in, it is important to thoroughly understand the uptake mechanisms in each cell type. However, NP uptake is not governed by a single mechanism; instead, it is a complex process influenced by several properties including their size, shape, surface charge, and functionalisation. Although tuning these properties can improve uptake, designing a NP that universally exhibits optimal uptake is far from trivial. In the case of cancer targeting, this is further complicated by inter-tumour heterogeneity – differences between tumour masses within a patient, or between different patients with the same cancer type – as well as intra-tumour heterogeneity, as solid tumours often contain a diverse array of cell types.

**Fig. 3:**
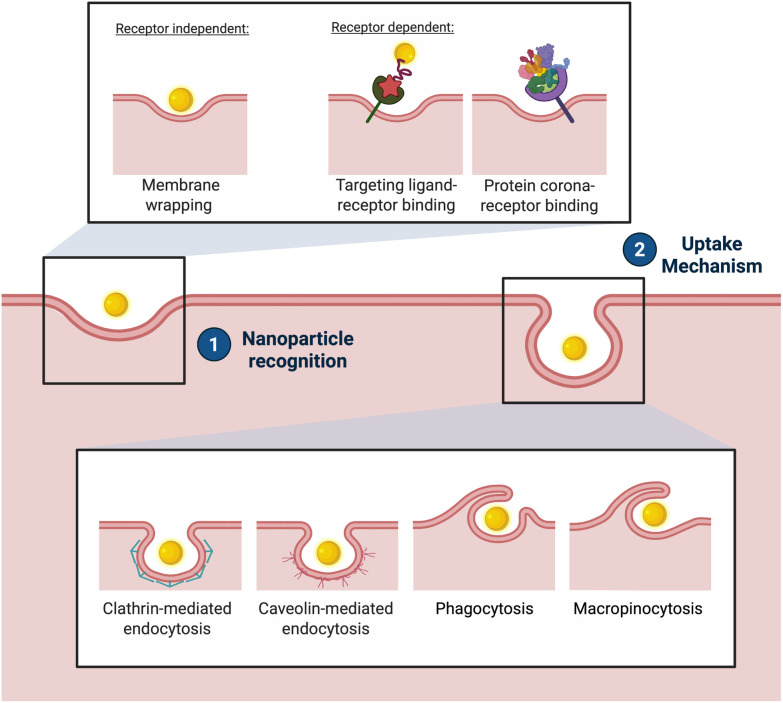
Nanoparticle uptake: (1) receptor independent and receptor dependent methods of NP recognition at the cell surface; (2) mechanisms of NP uptake.

The mechanisms underlying NP uptake (Fig. 3) have been investigated extensively, yet they remain poorly characterised for many types of NPs.^142^ Endocytosis pathways are believed to play a dominant role, including phagocytosis, macropinocytosis, and clathrin- or caveolae-mediated endocytosis.^143^ Among these, clathrin- and caveolae-mediated endocytosis are the most relevant for nanoscale structures, involving the uptake of molecules *via* vesicles coated in the protein clathrin, or 50–60 nm membrane invaginations called caveolae, respectively.^144^

Endocytosis of functionalised AuNPs can occur either through receptor-mediated pathways, *via* their target receptors, or non-specifically by interacting with the membrane. Studies have found that ligand-coated spherical NPs can enter and exit cells by membrane wrapping, even in the absence of clathrin or caveolin involvement.^145,146^ Conversely, other research suggests that endocytosis of certain AuNPs is dependent on clathrin-mediated pathways.^147^

Non-functionalised NPs can be internalised by cells without involving receptor mediation. Studies have identified three distinct size-dependent interactions between AuNPs and lipid membranes: (1) cooperative aggregation and wrapping through a membrane tube for NPs sized 5–10 nm, (2) adsorption and internalisation for NPs sized 25–35 nm, and (3) less likely adsorption for NPs sized 50–60 nm. It has been suggested that the minimum diameter required for spontaneous membrane wrapping of citrate-coated bare AuNPs is 4 nm.^148^ Interestingly, non-functionalised AuNPs can also be internalised *via* receptor-mediated endocytosis, due to the non-specific adsorption of serum proteins on their surface, the protein corona.^149^ Particles sized 25–30 nm were found to display the fastest uptake *via* receptor-mediated endocytosis.^147^ This highlights the interplay between NP size, surface properties, and cellular uptake mechanisms, even in the absence of specific functionalisation.

Phagocytosis, a major pathway used by immune cells to clear foreign bodies, is also important to consider, as NPs can be detected and taken up by macrophages throughout their journey. The extent to which they are sequestered significantly influences their circulation time and, consequently, the efficiency of their delivery to the disease site. Uptake by macrophages in the bloodstream or by tumour-associated macrophages (TAMs) within the tumour microenvironment is thought to contribute to the poor delivery of NPs to cancer cells.^118^ Again, the protein corona composition also plays a major role in immune recognition and thus macrophage phagocytosis of NPs, and its manipulation could allow enhanced tumour uptake. Ultimately, achieving a balance between efficient uptake in target cells and lower uptake in non-target cells is crucial for localising NPs to the disease site.

Although the canonical pathways of AuNP internalisation such as clathrin- and caveolae-mediated endocytosis are well established, recent studies have revealed additional layers of complexity in how particle properties govern cellular interactions. Evidence suggests that AuNPs can exploit noncanonical and energy-independent pathways, including direct membrane translocation or fusion-like mechanisms under specific physicochemical conditions.^150^ Moreover, chirality has emerged as a key determinant of uptake efficiency and downstream signalling, as demonstrated by the chirality-dependent uptake of gold nanooctopods^151^ and the distinct immune responses elicited by left- and right-handed AuNPs.^152^ Recent perspectives emphasise that progress in chiral nanostructures will depend on a deeper theoretical understanding of how multiscale chirality shapes optical, electronic, and biological behaviour.^153^ Together, these findings underscore a shift from static descriptions of NP uptake toward a more adaptive, structure- and context-dependent understanding of nano–bio interactions.

## 5. Microscopic imaging of gold nanoparticles

To visually investigate NP uptake and migration mechanisms and determine their precise localisation and distribution *in vitro*, sophisticated imaging techniques are needed. Given the nanoscale size of the particles, these techniques must provide extremely high magnification and exploit the unique optical properties of AuNPs to enhance visibility. Advanced imaging techniques have been developed to enable the direct visualisation of either single or clusters of AuNPs within biological systems. Although this is fairly easy on 2D models, imaging 3D models presents additional challenges, as most techniques lack the ability to penetrate deeply into tissues, often requiring sectioning, which is time-consuming and tedious.

### 5.1. Imaging gold nanoparticles using fluorophores

AuNPs can be visualised by fluorescence-based microscopy through the conjugation of fluorescent moieties on their surface. Such techniques include fluorescence light or confocal microscopy, laser scanning confocal microscopy,^154^ as well as techniques involving X-ray fluorescence^155^ and computed tomography.^156^ Although useful, fluorescent labelling presents several limitations. Some arise due to the intrinsic photochemistry of the labelling molecules, which can render them susceptible to photobleaching, photoblinking, optical saturation^157^ and incompatibility with aqueous environments.^158^ In the context of AuNP labelling, fluorophores can also undergo metal-induced quenching, which can be mitigated by introducing spacer layers or core–shell (*e.g.*, Au@SiO) designs.^159^ These factors restrict observation times and limit the speed of measurements. Additionally, modification with fluorescent labels can alter the nanoparticle's physicochemical properties, such as size, shape and charge, potentially introducing artefacts into result interpretation.^160,161^ To avoid such errors, rigorous characterisation and control experiments must be in place to validate findings and ensure consistency with the label-free versions. Since the NPs intended for clinical translation will be label-free, pre-clinical *in vitro* experiments should ideally also use label-free NP systems. Therefore, developing techniques that do not rely on external fluorescence, and nanomaterials compatible with them would be ideal. Metallic nanostructures are good candidates for such systems, as, under some conditions, they can even be intrinsically brighter than fluorophores.^162^ Nevertheless, despite the downsides of labelling and the label-free opportunities AuNPs offer, we have shown that 69% of recent AuNP studies looking at AuNP barrier crossing still use labelled AuNPs (Fig. 1B).

### 5.2. Imaging label-free gold nanoparticles

The interactions of gold with light have been recognised for over a century,^163^ with later studies revealing the unique optical properties exhibited by nanoscale gold as a result of localised surface plasmon resonance (SPR). This occurs when the free electrons on the surface of the AuNPs oscillate collectively upon interacting with electromagnetic waves.^164^ SPR is responsible for the large absorption and scattering cross-section of AuNPs, which have been harnessed in numerous label-free imaging techniques (Table 2).

**Table 2:** Techniques for imaging AuNPs ranked in order of decreasing suitability for label-free AuNP detection^44,157,167–186^

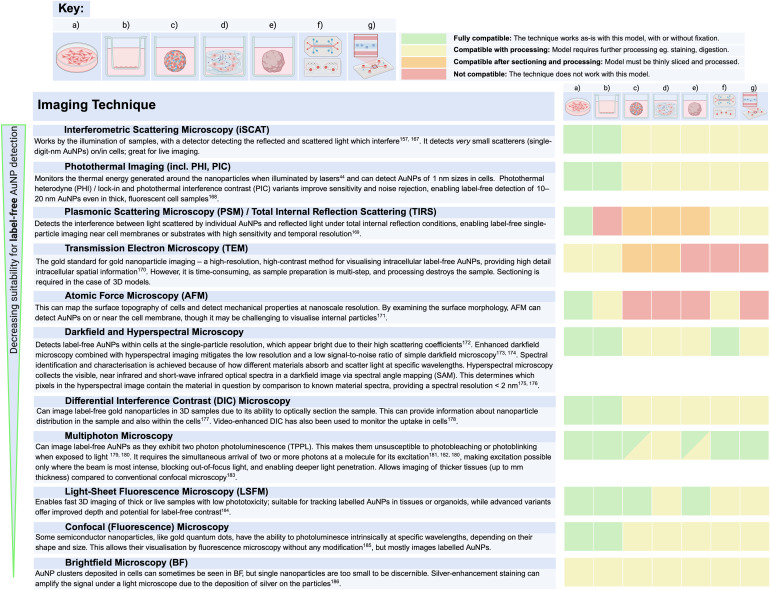

Such techniques are generally straight-forward when working with 2D models, but challenges arise with more complicated models. When observing 3D models, conventional fluorescence and confocal microscopy techniques are limited by penetration depth. These methods typically cannot image tissue regions deeper than about 200 μm, as most excitation light is absorbed or scattered before reaching deeper layers, limiting the detection of NP penetration. One way to overcome this is by thinly slicing the 3D model for imaging individual sections. Spheroid models or samples from *in vivo* studies are commonly cryosectioned^165,166^ or fixed and sliced using an ultramicrotome,^35^ producing micrometer- or nanometer-thick sections suitable for microscopy. This enables the detailed assessment of NP uptake and penetration in both peripheral and interior regions of the spheroids.^165^ However, this process is labour-intensive and time-consuming, particularly when working with the small size of spheroids, and results often rely on a few sample slices. Therefore, developing new methods to enable deeper light penetration for imaging intact 3D models is a critical area of ongoing research. For microfluidic systems, like organ-on-a-chip or microfluidic vascular networks, some of these techniques are suitable only if the systems consist of optically clear materials.

## 6. Quantifying gold NPs in cells

Considerable effort has also been dedicated to the advancement of techniques that can accurately and reproducibly quantify NPs in cells across multiple experimental settings (Table 3). However, no single quantification method is universally optimal; the most appropriate approach depends on the specific research question and the type of NPs used.^187^

**Table 3:** Techniques for quantifying AuNPs ranked in order of decreasing suitability for label-free AuNP detection^180,192–197^

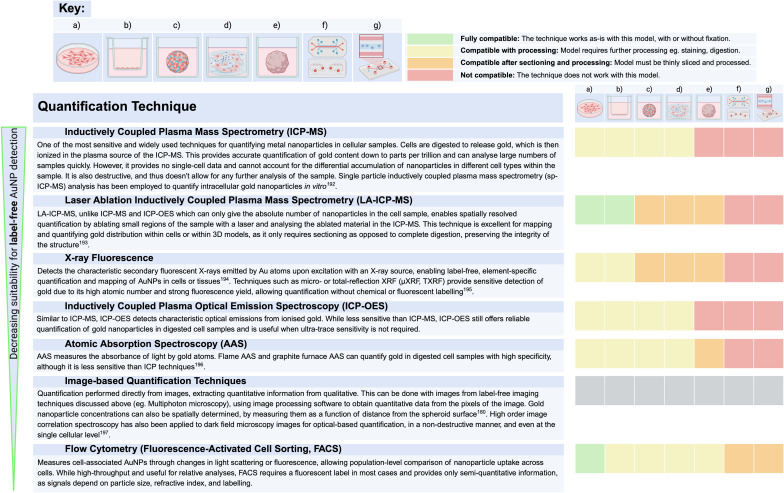

One of the major challenges in NP quantification, particularly when determining appropriate NP doses for therapeutic applications, is identifying the most relevant parameter to measure. Doses can be normalised by mass, surface area, or particle number, each of which provides a different perspective on NP characterisation.^187^ Although mass is the easiest to measure, it cannot fully capture the nature of NPs. Surface area accurately accounts for the surface reactions of functionalised NPs. However, a study that determined a general dose threshold (at 1 trillion NPs) for maximised NP delivery of 12% in mouse tumours, has shown that normalisation of this dose by surface area obscured the dose threshold.^188^ This suggests that particle number is perhaps the most representative parameter, especially when size distribution is taken into consideration.^189^

A key advantage of using AuNPs is the natural scarcity of gold in biological systems. Since normal cells do not contain any gold by nature, elemental analytical techniques can be used to quantify AuNPs by assessing the elemental composition of samples. Additionally, AuNPs offer a unique benefit in imaging-based quantification, as they may not require fluorescent labelling. Images obtained by label-free techniques discussed earlier could be directly used for quantification. There are also fluorescence-based techniques including fluorescence microscopy-based quantification and fluorescence assays, which do require external fluorescent moieties.^190,191^

A major limitation of most conventional quantification techniques is that they typically provide absolute NP counts in a sample, but offer no information about their location within it, unlike imaging-based approaches. Additionally, sample preparation for many of these techniques can be destructive and/or invasive, rendering the sample unusable for further analytical or imaging studies to determine spatial distribution. For example, NP quantification in 3D models often requires complete digestion of the model before the NPs can be extracted for quantification, resulting in permanent sample loss. Also, most techniques are unable to discriminate between intracellular NPs and cell surface-adherent NPs or those trapped in other components like the ECM.

Multiple imaging and quantification techniques can be used in concert to more accurately track NP movement, uptake, and interactions, providing a more comprehensive understanding of their behaviour.^198^ The selection of techniques depends on the available equipment and the impact of each method on the sample – whether it can preserve sample integrity to allow downstream analysis. Specifically, our systematic data extraction from recent papers investigating AuNP barrier crossing *in vitro* (Fig. 1) revealed that the most common combinations of imaging and quantification techniques were confocal microscopy with ICP-MS/ICP-OES and confocal microscopy with image-based quantification (each representing 36% of studies), followed by TEM with ICP-MS/ICP-OES (11%). Although these techniques are widely available and thus remain prevalent in *in vitro* NP studies, some lack the sensitivity required for label-free AuNP detection and fail to capture the multidimensional spatiotemporal dynamics governing nanoparticle transport across biological barriers.

Recently, imaging and quantification approaches have also been augmented through the integration of artificial intelligence (AI), which can aid in the automated detection and analysis of AuNPs. Deep learning models have been successfully applied to identify and segment AuNPs in TEM micrographs of tumour cells, achieving accuracies comparable to expert annotation while reducing analysis time.^199^ Similarly, AI-assisted light-sheet microscopy pipelines now enable quantitative, organ-scale mapping of nanoparticle deposition in complex 3D tissues and organoids, improving the fidelity and throughput of biodistribution assessments.^200^ In hyperspectral imaging of label-free AuNPs, ML approaches have been employed to distinguish between background noise and overlapping particles,^201^ and ML–based spectral unmixing could used to distinguish AuNP scattering or plasmonic signals from autofluorescence or background noise, as typically done in fluorescence microscopy,^202^ enhancing signal specificity. Beyond image interpretation, AI-driven data fusion frameworks combining imaging and ICP-MS outputs are emerging to correlate spatial and quantitative information automatically, offering a path toward reproducible, multimodal NP analysis.^203^

## 7. Conclusions

NPs, and particularly AuNPs, hold considerable promise as a theranostic tool, hinting at a future where targeted therapeutic and diagnostic approaches are seamlessly integrated. However, realising this potential requires further research to elucidate the mechanisms governing NP uptake and transcellular migration. *In vitro* models provide a powerful platform for testing NP movement across biological barriers, and with the correct imaging and quantification methods, a comprehensive understanding of their journey from the bloodstream to the disease site can be achieved. This highlights the need for continued exploration of advanced, non-invasive imaging and quantification techniques that can accelerate biological studies involving NPs. Importantly, the development of techniques that enable the observation and quantification of label-free NPs is important to ensure that *in vitro* results accurately reflect the behaviour of the NPs intended for human administration, without the presence of labelling molecules that may alter the NP properties. AuNPs can serve as a good NP model for these studies due to their physicochemical profile which makes them compatible with such techniques.

Despite the slow development and translation of AuNP-based therapies, hope is far from lost, with many research groups and experts around the world committed to overcoming these challenges to develop safe and effective therapies. The multifunctionality of these particles means that the potential rewards from these studies are vast. Overcoming the challenges that hinder the translation of AuNP formulations could unlock numerous novel therapeutic and diagnostic approaches in the clinic. Therefore, as the chemical innovation and biological studies continue to converge, AuNPs could enable the evolution of targeted therapies which are poised to revolutionise modern medicine.

## Author contributions

C. C.: Conceptualization, investigation, writing – original draft, visualization, and literature search. A. J. M.: Supervision, investigation, writing – review & editing. M. L.: Supervision, project administration, writing – review & editing. B. R.: Supervision. K. R.: Supervision, investigation. C. T.: Writing – review & editing. G. J.: Supervision, investigation, writing – review & editing. H. P.: Supervision, investigation, writing – review & editing.

## Conflicts of interest

There are no conflicts to declare.

## Data Availability

No primary research results, software or code have been included and no new data were generated or analysed as part of this review.
